# Hot-Volumes as Uniform and Reproducible SERS-Detection Enhancers in Weakly-Coupled Metallic Nanohelices

**DOI:** 10.1038/srep45548

**Published:** 2017-03-30

**Authors:** José M. Caridad, Sinéad Winters, David McCloskey, Georg S. Duesberg, John F. Donegan, Vojislav Krstić

**Affiliations:** 1 School of Physics and CRANN, AMBER Research Centre, Trinity College Dublin, College Green, Dublin 2, Ireland; 2 School of Chemistry and CRANN, AMBER Research Centre, Trinity College Dublin, College Green, Dublin 2, Ireland; 3 Department of Physics, Chair for Applied Physics, Friedrich-Alexander-University Erlangen-Nürnberg (FAU), Staudtstr. 7, 91058 Erlangen, Germany

## Abstract

Reproducible and enhanced optical detection of molecules in low concentrations demands simultaneously intense and homogeneous electric fields acting as robust signal amplifiers. To generate such sophisticated optical near-fields, different plasmonic nanostructures were investigated in recent years. These, however, exhibit either high enhancement factor (*EF*) or spatial homogeneity but not both. Small interparticle gaps or sharp nanostructures show enormous *EFs* but no near-field homogeneity. Meanwhile, approaches using rounded and separated monomers create uniform near-fields with moderate *EFs*. Here, guided by numerical simulations, we show how arrays of weakly-coupled Ag nanohelices achieve both homogeneous and strong near-field enhancements, reaching even the limit forreproducible detection of individual molecules. The unique near-field distribution of a single nanohelix consists of broad hot-spots, merging with those from neighbouring nanohelices in specific array configurations and generating a wide and uniform detection zone (“hot-volume”). We experimentally assessed these nanostructures via surface-enhanced Raman spectroscopy, obtaining a corresponding *EF* of ~10^7^ and a relative standard deviation <10%. These values demonstrate arrays of nanohelices as state-of-the-art substrates for reproducible optical detection as well as compelling nanostructures for related fields such as near-field imaging.

Detecting and identifying individual molecules is the ultimate target of any sensing or diagnostics technique^1^,^2^,^3^,^4^,^5^,^6^,^7^,^8^,^9^,^10^. Currently, optical detection of few and down to the single molecule level is possible using different conceptual techniques such as fluorescence^1^, photoluminescence^2^ or surface-enhanced Raman scattering (SERS)^3^,^4^,^5^,^6^,^7^,^8^,^9^,^10^. Noteworthy, all of these methods rely on a common ground: the enhanced near-fields of plasmonic nature encountered in metallic nanostructured substrates, effectively acting as robust signal amplifiers^1^,^2^,^3^,^4^,^5^,^6^,^7^,^8^,^9^,^10^. Thus, the finest design of generic plasmonic nanostructures for the optical detection of molecules in very low concentrations requires the ability to generate simultaneously an intense, homogeneous and reproducible near-field enhancement^3^,^7^,^9^.

The fabrication of metallic nanostructured substrates has considerably evolved with time since the discovery of near-field enhancements at rough silver surfaces^7^. Initial substrates were primarily based on localized optical near-fields (“hot-spots”) appearing in narrow (<10 nm) interparticle gaps or at sharp edges^6^,^8^. However, they typically exhibit low signal reproducibility^3^,^8^,^10^, limiting their usage for applications^3^,^7^,^10^. The fundamental reason for this irreproducibility was recently ascribed^10^ to the non-uniform excitation of analyte molecules exposed to the inhomogeneous near-field generated at narrow hot-spots present in metallic nanostructures^8^,^10^. Therefore, substrates for reproducible optical detection demand not only high *EF*, but also extended regions of homogeneous (uniform) and intense near-field strength^3^,^4^,^10^. Importantly, although our scope here is dedicated to highly enhanced and uniform SERS-detection, near-fields providing these characteristics are also required in other related techniques such as near-field imaging^9^.

Currently, the generation of near-fields satisfying such demanding conditions is attempted by regular arrays of rounded and conveniently spaced individual nanostructures (monomers)^3^,^11^,^12^. So far, there are two basic shapes studied to design monomer-based substrates: nanospheres^11^,^12^ and –rods^3^,^5^. Both nanostructures have broad hot-spots, however, their maximum near-field intensity enhancement is moderate even when placed in an array configuration^3^,^5^,^11^,^12^. Such enhancement values are still low to be appropriate for very low concentrations of analytes, in particular for the case of single-molecule detection^6^,^7^. Here, we demonstrate how arrays of weakly-coupled Ag nanohelices have a reproducible, uniform and high near-field enhancement, reaching SERS *EF* values which are sufficient for detection of molecules in low concentrations, even down to the single-molecule level for the here tested SERS-active molecule^6^,^7^. With these near-field qualities, these substrates represent the first monomer-based substrate able to reach the limit of single molecule detection. This is possible due to the unique near-field distribution of a single nanohelix consists of broad hot-spots, merging with those from the neighbouring nanohelices in specific array configurations and generating a wide, uniform a highly enhanced detection zone, which we refer here as “hot-volume”. We obtain a SERS *EF* of ~10^7^ in our fabricated arrays of metallic nanohelices and a relative standard deviation (*RSD*) <10%. These values validate these substrates as state-of-the-art substrates for highly enhanced and reproducible SERS detection^4^,^6^,^7^, as well as functional nanostructures for related fields such as near-field imaging^9^.

## Results

### Homogeneous near-field enhancement in metallic nanohelices

**Figure 1:**
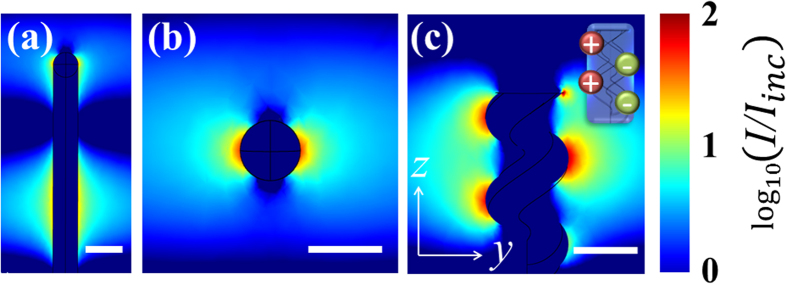
Near-field intensity enhancement of single Ag nanoparticles nanorod, nanosphere and nanohelix. (**a**) Near-field intensity of a single Ag nanorod on semi-infinite Ag substrate at 600 nm, close to the position of LSPR^3^. (**b**) Near-field intensity of a single Ag nanospere at 450 nm, close to the position of LSPR^27^. (**c**) Near-field intensity of a single Ag nanohelix on a semi-infinite Ag substrate at a wavelength of 650 nm, close to the position of their LSPR^13^. Inset shows the unique dipole distribution existing in individual nanohelices with charges from similar sign aligned on each side of the nanostructure^13^. All images correspond to cross-sections in the z-y plane and are plotted on a logarithmic scale for a better observation of the overall near-field distribution. All scale bars correspond to 100 nm. The hot-spot area mentioned in the main text corresponds to the area limited by plasmon penetration length (distance where the normalized electric field intensity falls to 1/*e* times of its maximum^24^).

**Figure 2:**
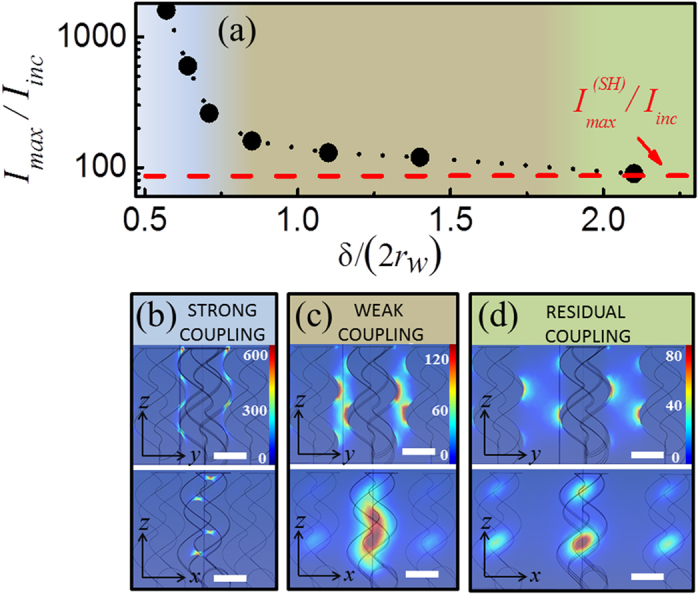
Near-field intensity of arrays of Ag nanohelices for different aspect ratios *δ*/(2*r*_*w*_). (**a**) Maximum (normalized) near-field intensity enhancement *I*_*max*_/*I*_*inc*_ of Ag nanohelices for different *δ*/(2*r*_*w*_). The dashed red line indicates the maximum near-field intensity enhancement of a single helix. Three different regimes can be depicted representing strong (blue), weak (brown) and residual (olive) interparticle coupling. (**b**) Near-field intensity distribution at an aspect ratio *δ*/(2*r*_*w*_) of 0.7 (strong coupling). (**c**) Near-field intensity distribution at an aspect ratio of 1.1 (weak coupling). (**d**) Near-field intensity distribution at an aspect ratio of 2.1 (residual coupling). The excitation wavelength in all cases is 650 nm as in Fig. 1c. All scale bars are 100 nm.

According to far-field studies, the optical response of a nanohelix is governed by localized surface plasmon resonances (LSPR)^13^,^14^,^15^,^16^,^17^,^18^,^19^,^20^. Therefore, regular metallic nanohelices bear the potential of converting incoming light into strongly enhanced and localized optical near-fields^18^. Figure 1 compares the calculated (see Methods) electric field intensity distribution of the commonly used nanorods and -spheres with the one of a nanohelix. At a wavelength close to the position of their LSPR, the ratio between the maximum near-field intensity from single nanohelices 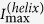
with respect to the incident radiation *I*_*inc*_ is ~85, a value which is more than three times higher than the value for nanorods^1^ (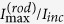 ~22) and is over twice that of the nanospheres^11^,^12^ (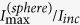 ~40). Particularly, in a single helix (Fig. 1c) three electric field antinodes appear per turn due to its longitudinal LSPR^13^,^20^ (that is, two consecutive dipoles per turn Fig. 1c, inset). Each one of these ‘hotspots’ cover a large area of ~100 nm in length and a plasmon penetration depth of ~40 nm. Furthermore, multiple-turn nanohelices^13^ have a noticeable near-field enhancement of ~10% (turquoise colour, Fig. 1c) due to dipole-dipole interaction between different turns, which extends along the distance of one pitch of the helix, *p*. In addition, in this unique near-field distribution, periodic dipoles are formed with an effective length of half a pitch^13^,^20^, *p*/2 (Fig. 1c). Thus, surface charges of one sign are accumulated on a side of the nanohelix at integer (*n*) positions *np*; whereas the opposite charge is accumulated on the other side of the nanohelix at positions shifted by *p*/2: *p*(*n* + 1/2). This peculiar charge distribution plays a determining role for the intercoupling strength in arrays of individual metallic nanohelices separated by a distance *δ* (see discussions below). To assess this, we calculated (Fig. 2) the maximum near-field intensity enhancement *I*_max_/*I*_*inc*_of a nanohelix placed in an array for different ratios *δ*/(2*r*_*w*_), were *r*_*w*_ is the radius of the wire forming the nanohelix.

**Figure 3:**
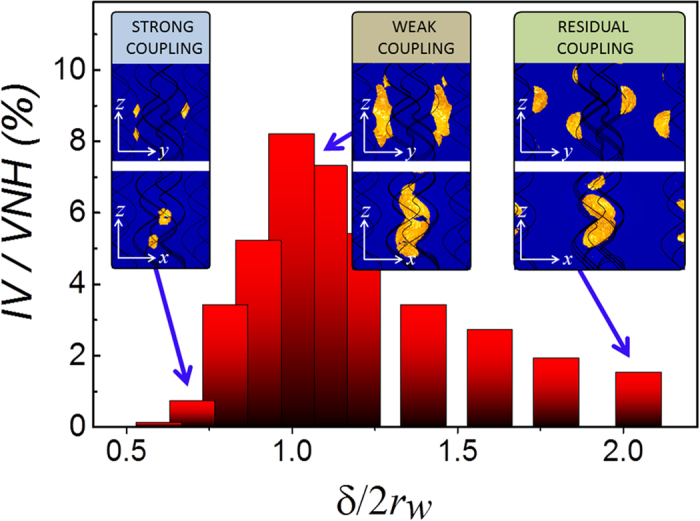
Hot-volume assessment in arrays of metal nanohelices for different aspect ratios *δ*/(2*r*_*w*_). Hot-volumes are estimated by the ratio of volume enclosed by the isosurface of (*1*/*e*)·*I*_*max*_*/I*_*inc*_ (*IV*) and the volume between neighbouring helices (*VNH*) in arrays of Ag nanohelices for different aspect ratios *δ*/(2*r*_*w*_). This ratio *IV*/*VNH* shows a clear maximum at *δ*/(2*r_w_*) ~ 1 (weak interparticle coupling) whereas it falls rapidly off when entering into the strong coupling regime (*δ*/(2*r_w_*) < 0.7). At this point, the *IV*/*VNH* ratio is ~8%, that is, 8% of the total volume between neighbouring nanohelices is filled by the isourface. Insets: (left) Visualization of the isosurfaces at 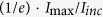 (orange) created in the strongly (*δ*/(2*r_w_*) = 0.7, left), weakly (*δ*/(2*r_w_*) = 1, middle) and residually (*δ*/(2*r_w_*) = 2.1, right) coupled regime.

While decreasing *δ*/(2*r*_*w*_), a significant continuous increase in *I*_max_/*I*_*inc*_ is found below *δ*/(2*r*_*w*_) ~ 0.7, attributed to a strong coupling regime^21^,^22^,^23^. Meanwhile, in the limit of large separations (*δ*/(2*r*_*w*_) > 2), *I*_max_/*I*_*inc*_ approaches the value of an individual nanohelix (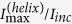) as expected for a residual coupling regime^21^,^22^,^23^. For intermediate values of *δ*/(2*r*_*w*_), a weakly interacting interparticle coupling regime exists. Figure 2b–d show representative near-field distributions for the three described regimes. In the strong and residual-coupling regimes only smaller and rather localised hot-spots are found. In contrast, in the weak coupling regime, large regions of high and uniform electromagnetic enhancement are generated, which, as aforementioned, we refer as “hot-volumes”. For an easier visualisation of these homogeneous enhanced near-field regions, the isosurface defining the plasmon penetration length^3^,^24^ at (1/*e*)·*I*_max_/*I*_*inc*_ from the near-field distributions in Fig. 2 are depicted in the insets of Fig. 3.

Importantly, this ability of the weakly coupled regime to generate hot-volumes stems from the exclusive (regular) hot-spot distribution along an individual nanohelix (Fig. 1c) allowing a unique interparticle coupling between neighbouring nanohelices at a distances *p*/2 (Fig. 2a, inset). For a better observation of the formation of the different hot-volumes generated when varying the interparticle distance, we plot (Fig. 3) the ratio between the volume enclosed by the isosurfaces at (1/*e*)·*I*_max_/*I*_*inc*_, *IV*, and the total volume existing between neighbouring helices *VNH* for several values of *δ*/(2*r*_*w*_). The histogram shows that there is a steady increase of *IV/VNH* coming from large interparticle distances peaking in the weakly coupled regime for *δ*/(2*r*_*w*_) ~ 1. Thereafter *IV/VNH* quickly falls off close to zero below *δ*/(2*r*_*w*_) ~ 0.7, that is when entering into the strong coupling regime. The combined results in Figs 2 and 3 underpin that indeed the generation of hot-volumes can be deliberately achieved by proper choice of the nanohelix’s parameters and their separations in an array. We further emphasise that the hot-volume at *δ*/(2*r*_*w*_) ~ 1 is larger than 3 × 10^5^ nm^3^ and therefore allows for the uniform excitation of even large biomolecules^3^. Moreover, this value comprises ~8% of the free space in between neighbouring nanohelices; exceeding considerably any reported volume fraction (<0.1% 10) in current substrates^3^,^4^,^5^,^6^,^7^,^8^,^9^,^10^,^11^,^12^. Importantly, weakly coupled nanohelices with *δ*/(2*r*_*w*_) between 0.8 and 1.1 possess similar *I*_max_/*I*_*inc*_ and normalized hot-volumes *IV/VN*, a fact suggesting robustness against fabrication tolerances of these nanostructured substrates^13^.

### Assessment of SERS detection in metallic nanohelices

**Figure 4:**
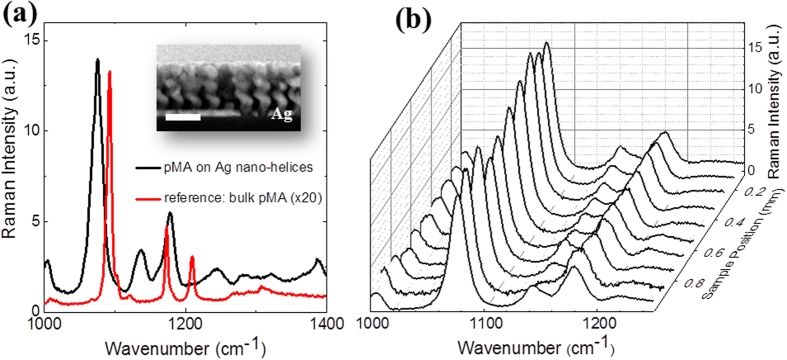
SERS of *p*MA. (**a**) SERS on Ag nanohelices compared to Raman on bulk-powder *p*MA. A clear enhancement of the Raman signal is observed in the Ag nanohelix array in comparison with bulk-powder *p*MA used as reference. Shifts in the positions of the peaks are observed as a result of the chemisorption of *p*MA^3^,^8^ on the surface of the nanohelices. Inset shows a scanning electron micrograph image of the fabricated Ag nanohelices^13^,^20^ (scale bar is 200 nm). (**b**) SERS spectra of *p*MA on a Ag nanohelix array for 10 different random positions along the length of the array (1 mm). We analyze commonly used peaks^3^,^8^ to calculate the relative standard deviation which lie at around 1080 cm^−1^ and 1180 cm^−1^.

To underpin our theoretical considerations on the use of arrays of weakly coupled nanohelices for optical detection of molecules in low concentrations, and thereby confirming their principle applicability, we fabricated^19^ a Ag nanohelix array with the aspect ratio of ~1 (Fig. 4a, inset). We opted for SERS as a commonly used optical technique for single molecule detection^3^,^4^,^5^,^6^,^7^,^8^,^9^,^10^ and measured their corresponding *EF* and *RSD* at 785 nm incident laser light with the widely used probing molecule^3^,^8^ *p*MA as analyte.

The choice of the 785 nm laser line is due to the position of the Ag nanohelix LSPR position at ~760 nm (Supplementary Note 4). After being fabricated, nanohelices were covered with the analyte by immersion into a diluted aqueous solution of *p*MA (see Methods). Through this, an adsorbed monolayer of these molecules forms on the nanostructured metal surface, as reported in the literature^3^,^8^.

Figure 4a shows the Raman spectra of *p*MA covering Ag nanohelix arrays and bulk-powder *p*MA used as reference. The presence of Ag nanohelices enhances considerably the Raman signal of the monolayer of *p*MA molecules deposited on their surface. The estimated *EF* of the present samples is at least 10^7^ as extracted from the two characteristic *p*MA Raman lines^3^,^8^ at around 1080 and 1180 cm^−1^ (*cf*. Methods and Supplementary Note 1). The main contribution is of electromagnetic origin as we have confirmed through additional measurements of *p*MA on an Ag thin film (Supplementary Note 1). Our observed *EF* is one order of magnitude larger than state-of-the-art, monomer-based, reproducible SERS substrates such as regular Ag nanorods^3^. The latter can be understood from both, the higher near-field intensity achieved by individual nanohelices with respect to nanorods (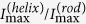 > 3), and the existence of a hot-volume rather than hot-spots in our samples. Furthermore, our *EF* compares to state-of-the-art, single-molecule SERS substrates with homogeneous near-fields, which, however, require additional filling (blocking) molecules^4^ to ease the inhomogeneity of the near-field of highly localised hot-spots for the analytes and bears the possibility of interference of the individual Raman responses^4^ of filling and analyte molecules. The performance of the arrays of nanohelices, however, does not involve any additional treatment.

Finally, we assessed the reproducibility of the SERS signals in our Ag nanohelix arrays measuring 10 random spectra separated ~100 μm across the entire sample area (1 mm × 100 μm). We obtained highly repeatable spectra (Fig. 4b) and estimated *RSD* values of ~10% and ~6% for the two aforementioned Raman lines (1080 and 1180 cm^−1^, respectively; *cf*. Supplementary Note 2). Again these values are within the range of state-of-the-art substrates^3^,^4^,^5^. We reconfirmed these *RSD* values by using graphene as an alternative probing molecule (Supplementary Note 2).

## Discussion

Altogether, the unique optical near-field of an individual nanohelix allows the creation of large and homogeneous near fields (hot-volumes) in arrays of nanohelices for aspect ratios *δ*/(2*r*_*w*_) ~ 1 via weak interparticle plasmon coupling. These volumes comprise up to 8% of the free space inbetween neighbouring nanohelices. By using SERS as a common optical detection technique and without any further optimization, these arrays can reach high *EF* ~ 10^7^ and *RSD* ≤ 10%, as exemplified on an array of Ag nanohelices. These values are superior to other (well separated) monomer-based substrates^3^,^5^, reaching the limit for reproducible single-molecule detection^6^,^7^ for the studied analyte (*p*MA). In addition, they are comparable to substrates containing narrow hot-spots with interfering filling molecules to achieve a homogeneous near-field^4^. More generally, the present results provide principle design strategies to achieve uniform, spatially extended and intense near-fields via weak interparticle coupling of plasmonic nanostructures. Thus, these systems are not only suitable for molecular detection and analytics as demonstrated here, but also for other related near-field techniques such as fluorescence^1^ or tip-enhanced Raman spectroscopy (TERS) imaging^2^,^9^.

## Methods

### Fabrication of metallic nanohelices

Periodic arrays of regular metallic nanohelices are engineered at the nanoscale by oblique angle deposition on rotating substrates^19^. For the present study (Supplementary Information), we fabricated arrays of Ag nanohelices (size 1 mm × 100 μm) keeping for each array similar helical parameters (pitch *p* ~ 128 nm, diameter *D* ~ 75 nm, number of turns *N* ~ 2.8, and wire radius *r*_*w*_ ~ 35 nm). The centre-to-centre distance between nanohelices is 150 nm, thus the separation distance between helices is *δ* ~ 75 nm and the achieved aspect ratio is *δ*/(2*r*_*w*_) ~ 1. Samples were kept under vacuum after fabrication prior to the Raman measurements.

### Measurements and determination of EF

Raman spectra acquisition was done at ambient conditions in a Renishaw micro-Raman spectrometer using a ×50 objective lens. The laser wavelength used was 785 nm. The calibration was carried out with the aid of the Rayleigh and Si bands at 0 cm^−1^ and 521 cm^−1^, respectively. The micro-Raman resolution is ~2 cm^−1^. Acquisition time was 1 min, and the laser power was 1 mW. Experimentally, the *EF* can be determined by the expression^3^,^4^,^5^,^6^,^7^ 
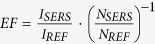
. *EF* represents the ratio of the SERS signal *I*_*SERS*_ to the reference Raman signal, *I*_*REF*_, normalised per molecule, where *N*_*SERS*_ and *N*_*REF*_, respectively, are the number of molecules giving rise to the Raman response with SERS and on a reference substrate.

Regarding the *EF* determination, we calculate the number of *p*MA molecules in the bulk-powder reference sample *N*_*REF*_ and in our nanostructured substrate *N*_*SERS*_ as follows: we have 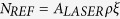 where *ξ* is the focal depth of the laser spot (12 μm for 785 nm wavelength), *ρ* ~ 6.5 × 10^27^ m^−3^ (1.18 g cm^−3^) is the density of *p*MA molecules^25^ and *A*_*LASER*_ is the area of the local spot of the laser. However, in our case there is no need to further estimate *A*_*LASER*_ since *N*_*SERS*_ depends on this variable, too, through^25^ 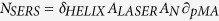 where 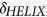
is the density of nanohelices (one nanohelix per surface of 150 nm × 150 nm), 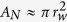 is the nanohelix footprint area, *r*_*w*_ is the radius of the wire composing the nanohelix, and 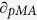 ~ 5 nm^−2^ is the surface density of absorbed *p*MA molecules^25^. One finds therefore 
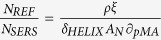
. Extracting *I*_*SERS*_ and *I*_*REF*_ from the experimental Raman *p*MA peaks at ~1080 cm^−1^ and ~1180 cm^−1^, we find an overall *EF* ~ 0.9 × 10^7^. We find the same *EF* magnitude also when using *p*MA on a thin film of Ag as reference (Supplementary Note 1).

### *p*MA thin layer on nanohelices

We exposed the Ag nanohelices for ~12 hours to an aqueous solution (10^−5^ M) of *p*MA. Through this we achieved a monolayer of these molecules attached to the nanohelices’ surface due to the common chemisorption process^3^,^8^ of *p*MA to silver.

### Numerical simulations

We simulate the near-field intensity of individual and arrays of nanohelices using the commercial finite element modelling (FEM) package, COMSOL (Supplementary Note 3). In all the cases, and adaptive mesh was used with maximum element sizes of 5 nm and a perfect conductor (PC) boundary condition in the plane z = 0 is used. The latter condition models the metallic Ag thin film existent in our samples underneath the nanohelices^19^. Before applying this PC approximation a comparison was made by simulating the full experimental substrate (50 nm silver, 300 nm SiO_2_ and Si base wafer); no differences were observed.

Light is incident along the negative direction in the z axis. The helices used have a pitch of 130 nm, a diameter of 75 nm and a diameter of the helical wire of 70 nm. All these values are close to those of the experimentally fabricated nanohelices. The scattered field is absorbed by perfectly matched layers (PML) regions. For all (individual and arrays) of nanohelices, the simulation domain is enclosed by five PMLs. Five elements were needed across the PML region to accurately absorb scattered field with less than 1% back reflection. In the case of arrays of nanohelices, we estimated the intercoupling effect in helical arrays by taking into account the contribution from the four nearest neighbours at different particle separation distances *δ*. Finally, the optical properties of the nanohelices are defined through the real and imaginary components of the refractive index^26^.

## Additional Information

**How to cite this article:** Caridad, J. M. *et al*. Hot-Volumes as Uniform and Reproducible SERS-Detection Enhancers in Weakly-Coupled Metallic Nanohelices. *Sci. Rep.* **7**, 45548; doi: 10.1038/srep45548 (2017).

**Publisher's note:** Springer Nature remains neutral with regard to jurisdictional claims in published maps and institutional affiliations.

## Supplementary Material

Supplementary Information

## References

[b1] Kinkhabwala A. . Large single-molecule fluorescence enhancements produced by a bowtie nanoantenna. Nature Photonics 3, 654–657 (2009).

[b2] Kallel H. . V. Photoluminescence enhancement of silicon nanocrystals placed in near-field of a silicon nanowire. Phys. Rev. B 88, 081302(R) (2013).

[b3] Huang J. A. . Ordered Ag/Si nanowires array: wide-range surface-enhanced Raman spectroscopy for reproducible biomolecule detection. Nano Lett. 13, 5039–5045 (2013). 24074380 10.1021/nl401920u

[b4] Chen H. Y., Lin M. H., Wang C. H., Chang Y. M. & Gwo S. Large-scale hot spot engineering for quantitative SERS at the single-molecule scale. JACS 137, 13698–13705 (2015). 10.1021/jacs.5b09111 26469218

[b5] Liu X., Shao Y., Tang Y. & Yao K.-F. Highly uniform and reproducible surface enhanced Raman scattering on air-stable metallic glassy nanowire array. Sci. Rep. 4, 5835 (2014). 25060646 10.1038/srep05835 PMC5376157

[b6] Lim D.-K. . Highly uniform and reproducible surface-enhanced Raman scattering from DNA-tailorable nanoparticles with 1-nm interior gap. Nature Nanotech. 6, 452–459 (2011). 10.1038/nnano.2011.79 21623360

[b7] Radziuk D. & Moehwald H. Prospects for plasmonic hot spots in single molecule SERS towards the chemical imaging of live cells. Phys. Chem. Chem. Phys. 17, 21072–21093 (2015). 25619814 10.1039/c4cp04946b

[b8] Hatab N. A. . Free-standing optical gold bowtie nanoantenna with variable gap size for enhanced Raman spectroscopy. Nano Lett. 10, 4952–4955 (2010). 21090585 10.1021/nl102963g

[b9] Johnson T. W. . Highly reproducible near-field optical imaging with sub-20-nm resolution based on template-stripped gold pyramids. ACS Nano, 6, 9168–9174 (2012). 22938087 10.1021/nn303496g

[b10] Fang Y., Seong N. H. & Dlott D. D. Measurement of the distribution of site enhancements in surface-enhanced Raman scattering. Science 321, 388–392 (2008). 18583578 10.1126/science.1159499

[b11] Wang C. X. . Preparation of nanoscale Ag semishell array with tunable interparticle distance and its application in surface-enhanced Raman spectroscopy. J. Phys. Chem. C. 130, 5523–5529 (2008).

[b12] Lal S. . Tailoring plasmonic substrates for surface enhanced spectroscopies. Chem. Soc. Rev. 20, 2527- 2533 (2008). 10.1039/b705969h 18443675

[b13] Caridad J. M., McCloskey D., Rossella F., Bellani V., Donegan J. F. & Krstić V. Effective Wavelength scaling of and damping in plasmonic helical antennae. ACS Photonics 2, 675–679 (2015).

[b14] Larsen G. K., He Y., Wang J. & Zhao Y. Scalable fabrication of composite Ti/Ag plasmonic helices: controlling morphology and optical activity by tailoring material properties. Adv. Optic. Mater. 2, 3, 24–249 (2014).

[b15] Kuzyk A. . DNA-based self-assembly of chiral plasmonic nanostructures with tailored optical response. Nature, 483, 311–315 (2012). 22422265 10.1038/nature10889

[b16] Mark A. G., Gibbs J. G., Lee T. C. & Fischer P. Hybrid Nano colloids programmed three-dimensional shape and material composition. Nature Mat. 12, 802–807 (2013). 10.1038/nmat3685 23793159

[b17] Esposito M. . Nanoscale 3D Chiral Plasmonic Helices with Circular Dichroism at Visible Frequencies. ACS Photonics 2, 105–114 (2015).

[b18] Schäferling M., Yin X., Engheta N. & Giessen H. Helical plasmonic nanostructures as prototypical chiral near-field sources. ACS Photonics 1, 6 530–537 (2014).

[b19] Caridad J. M., McCloskey D., Donegan J. F. & Krstić V. Controllable growth of metallic nanohelices at room temperature conditions. Appl. Phys. Lett. 105, 233114 (2014).

[b20] Zhang Z. Y. & Zhao Y. P. The visible extinction peaks of Ag nanohelixes: A periodic effective dipole model. Appl. Phys. Lett. 98, 083102 (2011).

[b21] Jain P. K., Huang W. & El Sayed M. On the universal scaling behaviour of the distance decay plasmon coupling in metal nanoparticles pairs: a plasmon ruler equation. Nanolett. 7, 2080–2088 (2007).

[b22] Ben X. & Park S. H. Size Dependence of the plasmon ruler equation for two dimensional metal nanosphere arrays. J. Phys. Chem. C. 115, 15915–15926 (2011).

[b23] Arias Castro J. C. & Camacho Beltrán A. S. Surface plasmon resonance of a few particles linear arrays. J. Electrom. Anal. Appl. 3, 458–464 (2011).

[b24] Maier S. A. Plasmonics: fundamentals and applications. Springer: New York (2007).

[b25] Hu X., Wang T., Wang L. & Dong S. Surface-Enhanced Raman Scattering of 4-Aminothiophenol Self-Assembled Monolayers in Sandwich Structure with Nanoparticle Shape Dependence: Off-Surface Plasmon Resonance Condition. J. Phys. Chem. C 111, 6962 (2007).

[b26] Palik E. D. Handbook of optical constants of Solids1^st^ ed. Academic Press (1997).

[b27] Mock J. J., Barbic M., Smith D. R., Schultz D. R. & Schultz S. Shape effects in plasmon resonance of individual colloidal silver nanoparticles. J. Chem. Phys. 116, 6755 (2002).

