# Supplementary material for: Hot-Volumes as Uniform and Reproducible SERS-Detection Enhancers in Weakly-Coupled Metallic Nanohelices
Source: Sci Rep. 2017 Mar 30;7:45548. doi: 10.1038/srep45548 (PMC5372087; doi:10.1038/srep45548)
Supplement: Supplementary Information [file srep45548-s1.pdf]

SUPPLEMENTARY INFORMATION

Hot-Volumes as Uniform and Reproducible SERS-Detection Enhancers in Weakly-Coupled Metallic Nanohelices

*José M. Caridad[1], Sinéad Winters[2], David McCloskey[1], Georg S. Duesberg[2], John F. Donegan[1] and Vojislav Krstić[1,3]**

[1]School of Physics and CRANN, AMBER Research Centre, Trinity College Dublin, College Green, Dublin 2, Ireland

[2]School of Chemistry and CRANN, AMBER Research Centre, Trinity College Dublin, College Green, Dublin 2, Ireland

[3]Department of Physics, Chair for Applied Physics, Friedrich-Alexander-University Erlangen-Nürnberg (FAU), Staudtstr. 7, 91058 Erlangen, Germany

*corresponding author: vojislav.krstic@fau.de

Supplementary note 1: SERS *EF* measurements and estimations

In the case of *p*MA, the estimation of the enhancement factor contribution due to a chemical or electromagnetic mechanism is unknown when using bulk-powder *p*MA as reference[1, 2]. This enhancement is shown clearly in Fig. S1, when comparing the Raman spectra of a *p*MA monolayer on Ag nanohelices with respect of bulk *p*MA. To distinguish between electromagnetic enhancement and chemical enhancement[2], we considered not only bulk *p*MA as reference but also *p*MA absorbed on evaporated thin silver films (inset, Fig S1.). As shown

below, the *EF* estimations for Ag nanohelices using both references indicate that the main part of the SERS *EF* is due to electromagnetic enhancement.

Figure S1

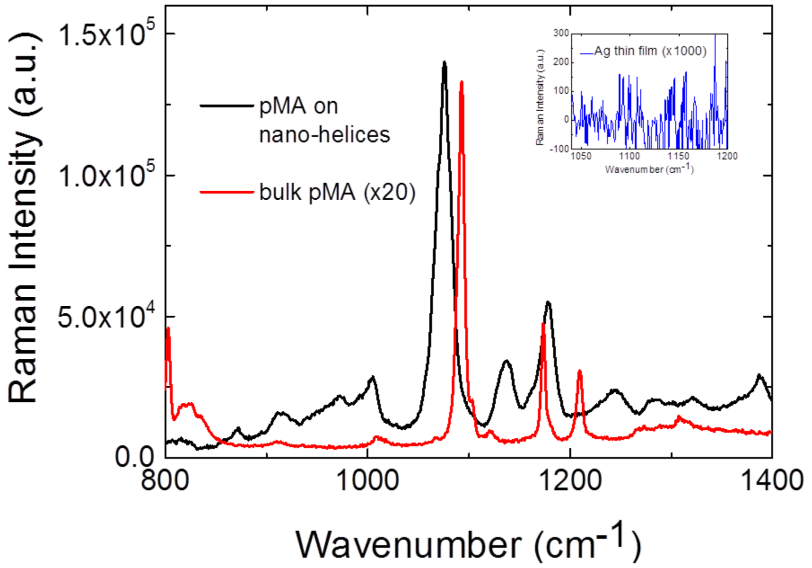

Figure S1. Raman spectra of bulk-powder *p*MA (red) and *p*MA on Ag nanohelices (black). Shifts in the positions of the peaks and different enhancements for both samples are observed as the result of the chemisorption process. Inset shows the Raman spectra of *p*MA on an Ag thin film; no Raman signal was detected.

<u>*EF estimation with Ag film reference substrate*</u>

In this case:

$$N_{REF} = A_{LASER} \partial_{pMA} \quad (1)$$

$$N_{SERS} = \delta_{HELIX} A_{LASER} A_N \partial_{pMA} \quad (2)$$

With this reference substrate, the ratio $\frac{N_{REF}}{N_{SERS}} = \frac{1}{\delta_{HELIX} A_N}$ is independent of A_{LASER} and ∂_{pMA}. In addition, the chemical enhancement component cancells out. I_{SERS} is obtained through the integration of the most prominent Raman *p*MA peak at ~ 1080 cm^{-1} and I_{REF} is

taken to be the equal to 0.2 (spectral noise), since within our set-up we did not detect any Raman peak of the *p*MA molecule (inset Fig. S1, inset). The estimated *EF* is then at least ~ 6 $\times 10^6$. We emphasize that this estimation provides only the electromagnetic *EF* of the helical nanostructures, not accounting for any possible chemical enhancement[2]. In other words, the main contribution to the SERS *EF* of Ag nanohelices is of electromagnetic origin.

Supplementary note 2: SERS reproducibility using graphene

The homogeneity and reproducibility of the spectra in Ag nanohelices using graphene as a probing molecule placed on top of the nanostructures was checked by measuring 10 different random spectra separated ~100 μm across the entire surface of the samples (100μm x 1mm) as showed in Fig. S2. We monitored the two main peaks of this monolayer material[4], the so-called G and 2D peaks, obtaining a relative standard deviation (*RSD*) of 9.6% and 5% for the G and 2D peaks, respectively. These values are similar to those reported in the case of *p*MA (see main text).

Figure S2.

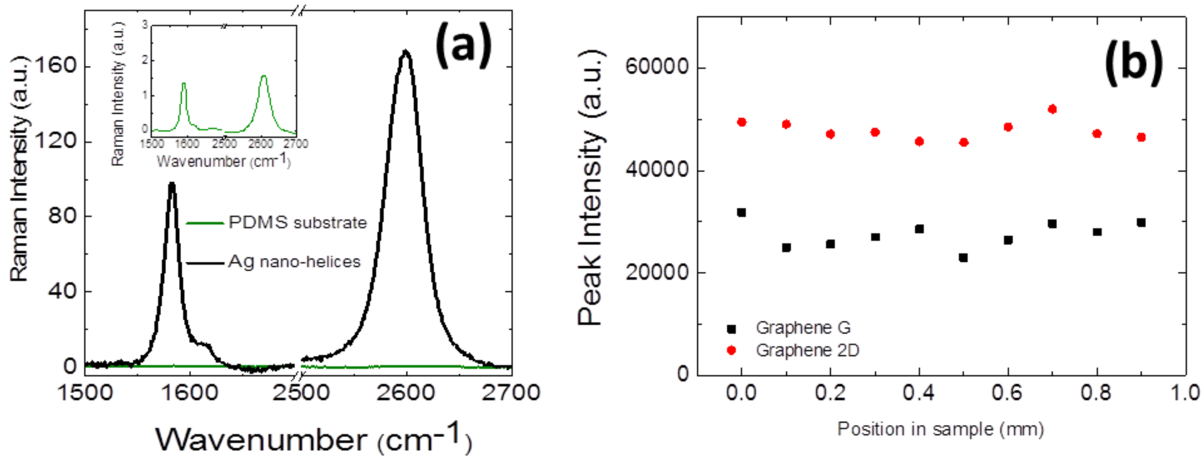

Figure S2. (a) Raman spectra of graphene on Ag nanohelices (black) and a reference substrate, polymethyl disiloxane, PDMS (green). Inset shows the G and 2D Raman peaks of graphene on PDMS (b) Peak intensity of G peak in graphene (black points) and 2D peak in

graphene (red points) on Ag nanohelices for 10 different positions along the 1mm long nanohelix array.

Supplementary note 3: FEM simulations

Estimation of near-field distribution and hot volume (normalized with respect to the volume between neighbouring helices) for arrays of Ag nanohelices

We simulate the near-field intensity of individual nanohelices and nanohelix arrays using the commercial finite element modelling (FEM) package, COMSOL. Light is incident along the negative direction in the z axis. The Ag helices used have a pitch of 130 nm, a diameter of 75 nm and a diameter of the helical wire of 70 nm. All these values are close to those of the the experimentally fabricated nanohelices. The scattered field is absorbed by PML regions. The simulation domain is enclosed by five PMLs. Five elements were needed across the PML region to accurately absorb scattered field with less than 1% back reflection. We first calculated the near-field intensity enhancement of Ag helical arrays by taking into account the contribution from the four nearest neighbours at different particle separation distances δ (see Fig. 2, main text) using the geometry depicted in Fig. S3. The optical properties of the nanohelices are defined through the real and imaginary components of the refractive index[5].

Figure S3

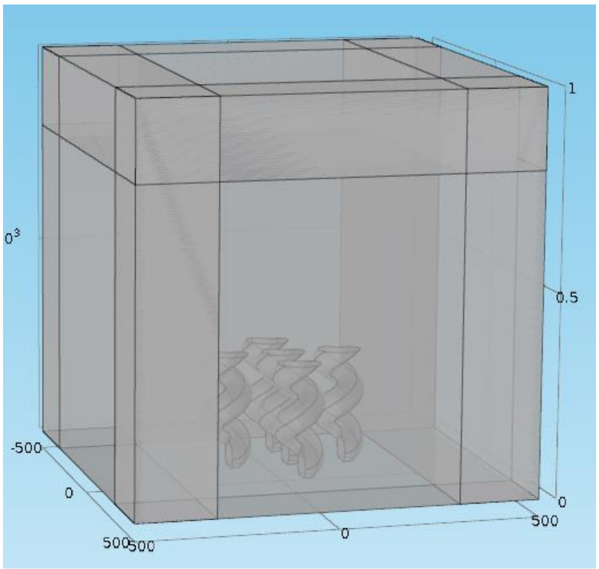

Figure S3. Geometry used for the simulations, considering the four nearest neighbours of a helix.

The calculation of the hot volume is carried-out by numerical integration of the volume of elements where the condition $\left(|E_{loc}|/|E_{inc}|\right)^2 \geq I_{\max}/I_{inc}e$ is satisfied. This hot-volume is delimited by an isosurface fulfilling the condition $\left(|E_{loc}|/|E_{inc}|\right)^2 = I_{\max}/I_{inc}e$, as depicted in Fig. 3d, main text. In addition, the hot-volume *HV* can be normalized (Fig.3 e) with respect to the free volume existent inbetween neighbouring helices, VNH (blue colour in Fig. S4). The quantity *HV/VNH* is therefore an indicator of how homogeneous is a SERS substrate.

Figure S4

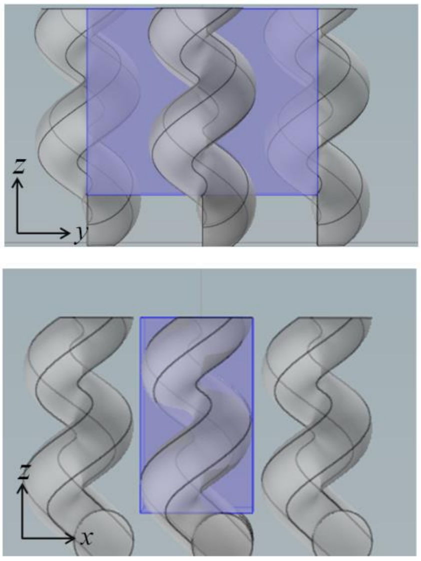

Figure S4. Free volume existing in between nanohelices (blue).

Supplementary note 4: Far-field reflectance measurements

Far-field reflectance measurements were carried out on our samples to extract experimentally the position where the longitudinal LSPR is located[6], thus the wavelength of maximum SERS enhancement [7]. However, we note how recent reports show no or little correlation between the positions where the LSPR maxima and SERS EF maxima occur[8]. In the latter case, our chosen wavelength would be a conservative estimation of the maximum SERS EF achieved by our Ag nanohelices.

The reflectance of helical nanostructures contains two main minima[6]. The one at higher wavelength (~2000 nm) indicates the effective medium threshold. For shorter wavelengths the reflectance is mainly due to the single particle response and the second minima appearing indicates the longitudinal LSPR wavelength λ_p [6] . Figure S5 shows the reflectance minima for Ag nanohelices around 750 nm. These values are in agreement with previous reports in the field[6].

Figure S5

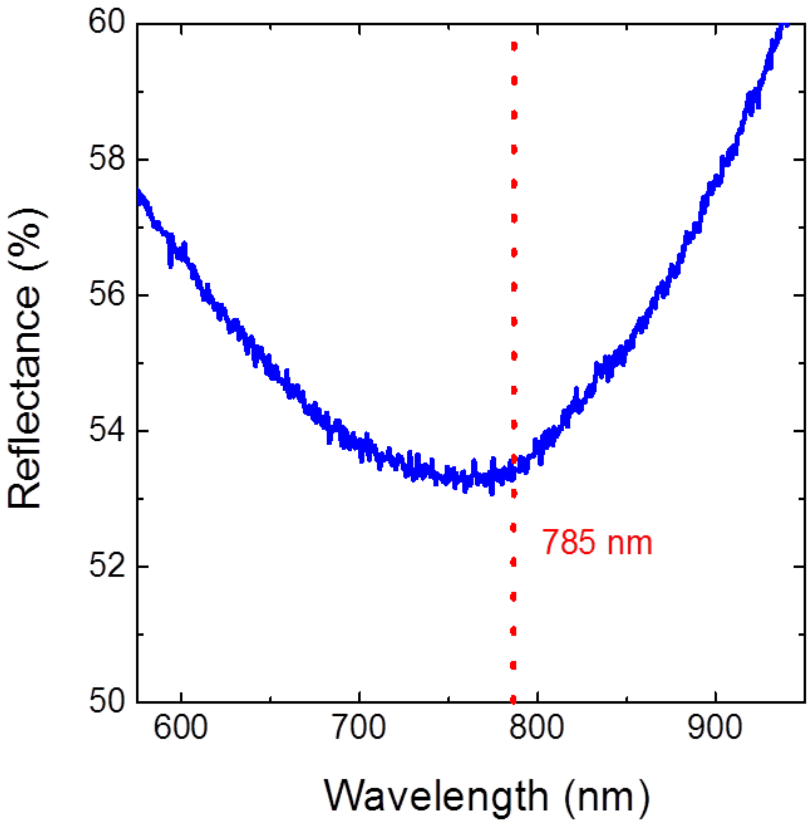

Figure S5. Reflectance measurements of Ag nanohelices used in the study (pitch p ~ 128 nm, diameter D ~ 75 nm, 3 turns, and wire radius ~35 nm) in the single particle wavelength-range[6]. Their minima is located around ~760 nm. Therefore, the LSPR is closer to the 785 nm laser wavelength used for the presented SERS measurements. Further discussions about reflectance measurements in metallic nanohelices, including analytical and numerical models, are found in Ref. 6.

Finally we point out that, while weakly coupled, the far-field response in our arrays of nanohelices with $\delta/(2r_w)$~1 can be predicted through the single particle behaviour. To justify this, using the universal “plasmon ruler equation”[9,10] we can estimate the maximum shift in the position of the longitudinal LSPR $\Delta\lambda_p$ due to interparticle coupling:

$\Delta\lambda_{\mathrm{p}} \sim 0.18 \cdot \lambda_p \exp\left(\frac{-[\delta/(2r_w)]}{0.23}\right)$. Thus, for our experimental case, $\delta/(2r_w)\sim 1$ and at wavelengths below 1000 nm, $\Delta\lambda_{\mathrm{p}}$ < 3nm. Furthermore, the extinction efficiencies of these weakly coupled systems are only <5% higher than the single particle response[9].

SUPPLEMENTARY REFERENCES

[1] Hatab, N. A. *et al*. *Nano Lett.* **10**, 4952-4955 (2010).

[2] Hu, X., Wang, T., Wang, L. & Dong, S. *J. Phys. Chem. C* **111**, 6962 (2007).

[3] Adar, F. *Spectroscopy* **25**, 16 (2010).

[4] Ferrari, A.C. *et al*. *Phys. Rev. Lett*. **97**, 187401 (2006).

[5] Palik, E. D. *Handbook of optical constants of Solids 1st ed*. (1997).

[6] Caridad, J. M., McCloskey, D. Rossella, F., Bellani, V., Donegan, J. F. & Krstić, V. *ACS Photonics* **2**, 675-679 (2015).

[7] Huang, J. A. *et al*. *Nano Lett*. **13**, 5039-5045 (2013).

[8] Kleinman, S. L. *et al. J. Am. Chem*. Soc. 13, 301 (2013)

[9] Jain, P. J., Huang, W. & El-Sayed, M. A. *Nano Lett.* **7**, 2080 (2007).

[10] Ben, X. & Park, S. H. *J. Phys. Chem. C*. **115**, 15915-15926 (2011).
